# Glial Cells in Spinal Muscular Atrophy: Speculations on Non-Cell-Autonomous Mechanisms and Therapeutic Implications

**DOI:** 10.3390/neurolint17030041

**Published:** 2025-03-13

**Authors:** Andrej Belančić, Tamara Janković, Elvira Meni Maria Gkrinia, Iva Kristić, Jelena Rajič Bumber, Valentino Rački, Kristina Pilipović, Dinko Vitezić, Jasenka Mršić-Pelčić

**Affiliations:** 1 Department of Basic and Clinical Pharmacology and Toxicology, Faculty of Medicine, University of Rijeka, Braće Branchetta 20, 51000 Rijeka, Croatia; tamara.jankovic@medri.uniri.hr (T.J.); iva.kristic@medri.uniri.hr (I.K.); jelena.rajic@medri.uniri.hr (J.R.B.); kristina.pilipovic@medri.uniri.hr (K.P.); dinko.vitezic@uniri.hr (D.V.); jasenka.mrsic.pelcic@uniri.hr (J.M.-P.); 2 Independent Researcher, 11741 Athens, Greece; 3 Department of Neurology, Clinical Hospital Centre Rijeka, Krešimirova 42, 51000 Rijeka, Croatia; valentino.racki@medri.uniri.hr

**Keywords:** spinal muscular atrophy, glial cells, neuroinflammation, synaptic dysfunction, myelination

## Abstract

Spinal muscular atrophy (SMA) is a neuromuscular disorder caused by homozygous deletions or mutations in the *SMN1* gene, leading to progressive motor neuron degeneration. While SMA has been classically viewed as a motor neuron-autonomous disease, increasing evidence indicates a significant role of glial cells—astrocytes, microglia, oligodendrocytes, and Schwann cells—in the disease pathophysiology. Astrocytic dysfunction contributes to motor neuron vulnerability through impaired calcium homeostasis, disrupted synaptic integrity, and neurotrophic factor deficits. Microglia, through reactive gliosis and complement-mediated synaptic stripping, exacerbate neurodegeneration and neuroinflammation. Oligodendrocytes exhibit impaired differentiation and metabolic support, while Schwann cells display abnormalities in myelination, extracellular matrix composition, and neuromuscular junction maintenance, further compromising motor function. Dysregulation of pathways such as NF-κB, Notch, and JAK/STAT, alongside the upregulation of complement proteins and microRNAs, reinforces the non-cell-autonomous nature of SMA. Despite the advances in SMN-restorative therapies, they do not fully mitigate glial dysfunction. Targeting glial pathology, including modulation of reactive astrogliosis, microglial polarization, and myelination deficits, represents a critical avenue for therapeutic intervention. This review comprehensively examines the multifaceted roles of glial cells in SMA and highlights emerging glia-targeted strategies to enhance treatment efficacy and improve patient outcomes.

## 1. Introduction

Spinal muscular atrophy (SMA), a rare autosomal recessive neuromuscular disorder, is caused by homozygous deletions or mutations in the survival motor neuron 1 (*SMN1*) gene, leading to insufficient SMN protein levels. The disease primarily affects lower motor neurons (MNs), resulting in progressive symmetrical muscle weakness and atrophy [1,2]. Its clinical spectrum is remarkably heterogeneous, ranging from prenatal onset with severe hypotonia and early mortality (Type 0) to adult-onset mild proximal weakness (Type 4) [3,4]. This phenotypic variability is largely modulated by the copy number of the *SMN2* gene, a partial compensatory “backup” gene, alongside other genetic and molecular contributors such as neuronal apoptosis inhibitory protein gene deletions [5].

The advent of disease-modifying drugs—including gene replacement therapy, antisense oligonucleotides, and splicing modifiers—has revolutionized SMA management. These therapies significantly improve survival, motor milestones, and quality of life, even in severe forms of the disease [3,4,6,7]. However, despite these advances, they do not fully address all aspects of SMA pathophysiology. Increasing evidence highlights the role of non-cell-autonomous mechanisms, particularly the involvement of glial cells, in disease progression. Glial cells, which include astrocytes, microglia, oligodendrocytes, oligodendrocyte progenitors, and radial glia, are essential components of the central nervous system (CNS). Beyond their classical roles in structural support, these cells regulate synaptic activity, maintain homeostasis, and respond to neural injury. Their dynamic interactions with neurons and each other are crucial for CNS function and are increasingly recognized as contributors to neurodegenerative disease pathology. Astrocytes, microglia, and oligodendrocytes play pivotal roles in maintaining MN function through neurotrophic support, calcium homeostasis, synaptic function, and inflammatory regulation. Their dysfunction exacerbates neurodegeneration by creating a toxic microenvironment, impairing synaptic stability, and amplifying inflammatory cascades.

Glial cell-targeted interventions represent a promising frontier for SMA therapy, complementing SMN-centric approaches [8]. For instance, targeting reactive astrogliosis or modulating microglial activation may mitigate neuroinflammation and improve neuronal resilience. Additionally, exploring pathways like the complement system, glutamate regulation, and sigma-1 receptor signaling offers novel therapeutic avenues. These strategies hold the potential to enhance MN survival, improve neuromuscular junction (NMJ) function, and prolong patient life expectancy.

This review aims to elucidate the multifaceted roles of glial cells in SMA pathophysiology and explore their therapeutic potential. By addressing gaps in current treatments and focusing on non-cell-autonomous mechanisms, this work seeks to contribute to the development of holistic and innovative approaches for managing SMA.

## 2. SMA as a Non-Cell-Autonomous Disease

Numerous studies have demonstrated that SMA is a cell-autonomous illness in which motor neuronal toxicity plays an important role. Recently, research has shifted towards glial cells and the inflammation they mediate. Glial cells have been shown to impact the condition’s progression and neuronal survival through various mechanisms [9].

Non-cell-autonomous pathways in neurodegeneration describe how damage or malfunction to one cell type can indirectly result in damage or death of another. These processes can worsen the symptoms and accelerate the course of the illness. In the context of SMA, astrocytic dysfunction is a critical example: astrocytes from SMA models exhibit impaired calcium homeostasis and reduced production of neurotrophic factors, which are essential for lower MN survival [10]. This disruption exacerbates lower MN vulnerability and contributes to the observed motor deficits. Such mechanisms underscore how metabolic deficiencies and altered cytokine signaling mediated by glial cells can create a toxic microenvironment for neurons, worsening disease progression [11]. The loss of the SMN protein in SMA leads to the selective degeneration of lower MNs in patients, a pattern also seen in SMA mouse models in vivo [9]. However, when lower MNs from SMN-deficient mice are cultured in vitro they do not exhibit increased cell death compared to healthy controls [12].

This contrasts with other neurodegenerative diseases, such as amyotrophic lateral sclerosis (ALS), where induced pluripotent stem cell-derived ALS lower MNs show significantly higher terminal deoxynucleotidyl transferase dUTP nick end labeling staining, indicating increased cell death by day 20 of differentiation compared to healthy controls [13]. Targeting SMN restoration only in lower MNs has a limited effect and suggests a role for non-cell-autonomous contributions to SMA MN vulnerability [14,15,16]. The benefit of *SMN1* restoration is not restricted to MNs. The prion protein promoter (PrP) targets neuronal cells, leading to high levels of SMN in neurons. This neuronal specificity is crucial for correcting the SMA phenotype, as muscle-specific SMN expression does not improve the condition. High levels of SMN expression driven by the PrP have been shown to rescue severe SMA mice. These mice, which would otherwise die shortly after birth, can survive for an average of 210 days with normal lower MN counts when SMN is expressed under the PrP. These findings indicate that SMA pathology involves other cell types, including astrocytes, sensory neurons, Schwann cells, and muscle cells, which may contribute to disease progression and lower MN loss [17,18,19].

Addressing non-cell-autonomous mechanisms, particularly those involving glial and peripheral cells, represents a critical frontier for developing more effective SMA therapies.

### Impaired Neurotrophic and Metabolic Support

Normally, upper and lower MN function is promoted by the secretion of important neurotrophic factors from astrocytes and microglia, including brain-derived neurotrophic factor (BDNF). It acts as a ligand and stimulates the Tropomyosin receptor kinase B (TrkB) and Ret receptors. By binding to the TrkB receptor, downstream pathways like the PI3K-Akt and Mitogen-Activated Protein Kinase (MAPK) become activated. At NMJs, TrkB signaling is essential for synaptic plasticity and the maintenance of neuromuscular transmission [20].

In SMA rodent models, there is evidence of reduced activation of the PI3K-Akt pathway. This occurs because of cytoskeletal defects that affect the localization and recycling of TrkB receptors at NMJs. This can lead to decreased sensitivity to BDNF and synaptic dysfunction, which is characterized by weak neuromuscular transmission, and which is presented by muscle weakness and atrophy observed in patients with SMA [21,22]. It has also been indicated that synaptic dysfunction occurs early in SMA, affecting both NMJs and central synapses before lower MN death. The impairment of synaptic integrity is linked to the loss of vesicular transporter 1-positive inputs to lower MNs, indicating a reduction in excitatory synaptic inputs [23].

The SMN protein deficiency in astrocytes has been shown to harm neighboring lower MNs. Some studies have indicated that a conditioned medium from SMA astrocytes leads to shorter neurites and reduced expression of MN-specific markers in cultured lower MNs [18,24]. Also, in vitro administration of peroxynitrite activates healthy astrocytes, enabling them to induce apoptosis in adjacent previously healthy lower MNs. This process occurs by releasing reactive oxygen species (ROS), which compromise neighboring cells by damaging membrane proteins, lipids, and oxidizing proteins essential for MN survival [25].

Recent evidence suggests astrocyte-secreted microRNAs may mediate non-cell-autonomous effects on lower MNs. One specific study has identified that astrocytes in SMA exhibit altered expression of microRNAs that can influence neuronal health. It has been found that miR-146a is upregulated in SMA astrocytes and induces lower MN loss when secreted into the extracellular environment [26]. Motor neuron cone growth, organization, and axonal prolongation show impairment in inadequate SMN protein levels [12,27]. As SMN protein is ubiquitously expressed in all human cells, including glia, studies have shown that SMN depletion specifically in lower MNs results in a less severe disease phenotype, indicating that glia may play a significant role in the progression of SMA [28,29]. Furthermore, the loss of SMN in all glia, in particular astrocytes and microglia, can result in their aberrant activation [30]. It has been detected that oxidative stress and the inflammatory response in the spinal cord microglia is regulated by SMN protein [31]. Also, restoration of *SMN1* under a prion promoter, highly expressed in neurons and astrocytes, showed a prolonged lifespan of SMA experimental animals [17].

Focusing solely on neuron-centered strategies in neurodegenerative pathologies has not led to significant discoveries which would change the understanding of the origin and the course of treatment of neurodegenerative diseases, including SMA [32]. The quad-partite synapse concept by Schafer et al. [33] explains how astrocytes, microglia, and neurons interact in the central nervous system (CNS) through secreted factors. This idea supports a broader perspective on brain communication. Due to the physiological inseparability of neurons and neuroglia in the homeostasis of the CNS, new therapeutic options for SMA need to investigate the role and therapeutic potential of astrocytes, microglia, oligodendrocytes, and Schwann cells in order to improve patient functions and prolong their lifespan [30].

## 3. Glial Cells in SMA: Types and Roles

Glial cells were first declared as connective tissue of the brain, “nervenkitt” (nerve glue), and termed as neuroglia [34]. Although glial cells are integral components of the CNS, they provide more than just structural and nutritional support. Glial cells are defined as radial glia, astrocytes, oligodendrocytes, oligodendrocyte progenitors, and microglia, with their numerous important functions depending on their origin and the interaction they have with their surrounding environment, including the interaction with neurons and each other [35]. They actively contribute to the functioning of the CNS to maintain homeostasis, modulate synaptic activity, and respond to injury. Numerous studies have highlighted their essential and complex roles, particularly in neurodegenerative diseases, such as SMA.

The human brain contains roughly an equal number of neurons and glia cells [35], and the number of each type of glia varies during human development and depending on the brain region [36,37]. Astrocytes are the most represented with around 20% of total adult brain cells [37], followed by oligodendrocytes with 25% [37], and microglia with 5–15% [38]. The least represented are oligodendrocyte progenitors, accounting for 3–10% [39] of total human brain cells [35].

Activation of neuroglia, mainly microglia and astrocytes, with accompanying infiltration of peripheral immune cells, initiates a process known as neuroinflammation, which occurs in numerous pathological entities of CNS and leads to neurodegeneration. A pathological characteristic of SMA is the formation of a glial bundle with concomitant loss of myelinated fibers [40], specifically in the areas of MNs degeneration, such as anterior roots and lumbar region [41]. Even though glia can limit neuronal loss and promote CNS regeneration, initiation of neurodegeneration can be stimulated by glia, mainly astrocytes and microglia [41].

Radial glial cells (RGCs) are derived from neuroepithelial cells and serve as progenitors for neurons, oligodendrocytes, and astrocytes [42,43]. RGCs are involved in the development of cortical layers of the human brain as they regulate direct or indirect production of both neurons and glia [35,44,45] and serve as guides for migrating neurons to their final positions in the brain [43]. During brain development, RGC fibers extend along the entire cortical wall and enable the migration of neuroblasts [46]. However, after the neuronal organization is complete, the aforementioned fibers disappear, and RGCs become astrocytes [47]. This differentiation of radial glia into astrocytes is regulated by various signaling pathways, including the MAPK/PI3K/SMAD pathways activated by transforming growth factor beta 1 [48]. It is considered that RGCs are not present in adult human brains; however, the exceptions are Bergmann glia in the cerebellum, Muller glia in the retina, and radial glia in the dentate gyrus of the adult hippocampus [49].

RGC dysfunction, such as dynein mutations, can affect lower and upper MN survival in SMA by disrupting neuronal migration, as RGCs guide neuroblasts during the brain and spinal cord development [50]. This disruption increases MN vulnerability. Furthermore, compromised RGCs may poorly differentiate into supportive astrocytes, reducing trophic support for MNs and worsening neurodegeneration. These impairments significantly contribute to SMA progression. Additionally, nuclear movement of RGCs require microtubule motor proteins such as dynein, and mutations of the tail domain of the heavy chain of cytoplasmic dynein (*DYNC1H1*) were identified to interrupt the formation and function of the dynein complex, which has been identified as a cause of SMA that has a lower extremity predominance [42,51,52] and of axonal Charcot-Marie-Tooth (CMT) disease [53]. Although the primary cause of SMA is SMN deficiency, recent research highlights a more extensive cellular dysfunction contributing to the disease’s pathophysiology, such as disruptions in microtubule dynamics in the activity of motor proteins such as dynein.

Cytoplasmic dynein’s (cytoplasmic dynein 1 and 2) are the molecular motor proteins involved in the retrograde transport of intracellular components and organelle positioning along microtubules [54,55]. In neuronal cells, dynein is considered as the primary molecular motor responsible for facilitating the movement of cellular components such as RNA granules, neurofilaments, vesicles, mitochondria, and signaling complexes [56]. Most studies focus on the cytoplasmic dynein complex, with *DYNC1H1* and its *DYNC2H1* mutations being linked to ciliopathies. Several mutations in the human *DYNC1H1* gene (I584L, K671E, Y970C) disrupt MN function, leading to progressive muscle degeneration and weakness [56]. The *DYNC1H1* mutations in human SMA are clinically observed congenitally or with an early onset with severe muscular weakness [48], and possibly in cortical malformations [42,57]. However, the lack of *DYNC1H1* mutations in cases of autosomal dominant SMA confirms that SMA is a genetically heterogeneous disease [51]. Bicaudal d (BCID) family adaptor proteins are a family of dynein regulatory proteins that control the velocity of dynein-based movements [54]. The published literature reports that mutations in the *BCID2* gene have been observed in patients with dominant congenital SMA with lower extremity predominance [58,59,60]. Although *BCID2* mutations are associated with dominantly inherited SMA, it is still uncertain whether they cause gain-of-function or dominant-negative loss-of-function effects.

### 3.1. Astrocytes: The Neurotrophic and Synaptic Regulators

Astrocytes are the most abundant cell type in the CNS and are characterized by their star-shaped look with numerous branching processes. They are regarded as essential support cells performing a wide range of complex functions. Based on cell morphology and anatomical location, they are classified into two subtypes: first, protoplasmic astrocytes, which are predominantly found in the gray matter, and feature numerous fine shoots alongside a few larger extensions; and second, fibrous astrocytes, located in the white matter, distinguished by their straight and long appendages [61,62]. Specialized forms of astrocytes, each associated with distinct anatomical regions, include Müller glia in the retina, Bergmann glia in the cerebellum, and tanycytes located at the base of the third ventricle [63].

Universal response of astrocytes to infection, trauma, or neurodegeneration is known as gliosis or reactive astrogliosis [64]. This process involves an increase in astrocyte number and/or size [42], alongside changes in gene expression and functional properties [63]. While reactive astrocytes can exhibit beneficial effects, such as promoting tissue repair [65], they may also contribute to pathological outcomes in conditions like brain [63] and spinal cord [66] trauma, ischemia, and autoimmune encephalomyelitis [63]. In SMA, reactive astrogliosis is consistently observed, particularly in regions of MN loss [42].

#### 3.1.1. Role of Astrocytes in Maintaining Synaptic Stability and Glutamate Regulation

Astrocytes form an interconnected network through gap junctions, facilitating continuous communication within the CNS. Additionally, they envelop blood vessels with their endfeet, playing a pivotal role in regulating metabolic balance, local blood flow, and the integrity of the blood–brain barrier [61]. Astrocytes play an active role in synaptic regulation by recycling neurotransmitters like glutamate, preventing its accumulation and subsequent cell death through “glutamate-mediated excitotoxicity” [67]. In performing this, they help maintain synaptic homeostasis and provide crucial support to neurons, particularly after brain tissue injury [68,69]. They also coordinate Ca^2+^ waves between neurons and astrocytes, aiding long-term potentiation. Communication between astrocytes and neurons is essential for complex neural networks. Finally, astrocytes interact with other glial cells, like microglia and oligodendrocytes, to maintain brain homeostasis [35].

#### 3.1.2. Dysfunction in Astrocyte-MN Interactions, Including Synapse Formation and Calcium Signaling

Astrocytes have been identified as integral components of “tripartite synapses”, alongside presynaptic and postsynaptic neurons [70]. They are essential for the formation, maturation, and plasticity of synapses, as well as neuron survival, through both diffusible factors and contact-mediated mechanisms. Among these, astrocytes secrete key molecules such as glial fibrillary acidic protein (GFAP), glial-derived neurotrophic factor (GDNF), and the pro-inflammatory cytokines interleukin-1β (IL-1β) and IL-6, which play a vital role in supporting the health and development of MN. These factors also interact directly with receptors on microglia, influencing the activity of microglia further [30,71]. In co-cultures of lower motor neurons and astrocytes, SMN deficiency disrupts synapse formation and function on motor neurons, likely through a contact-dependent mechanism. SMA astrocytes exhibit significantly lower Ephrin B2 expression than wild-type (WT) astrocytes, indicating impaired motor neuron–astrocyte interactions driven by Ephrin B2 downregulation, which may contribute to synapse formation deficits [72].

Calcium plays a crucial role as a second messenger, and astrocytes, unlike neurons that can generate action potentials, are electrically non-excitable. Instead, they rely on G-protein-coupled metabolic receptors to influence neuronal activity [73]. Consequently, Ca^2+^ signaling is commonly used as an indicator of astrocyte reactivity. Astrocyte intracellular calcium signaling is essential for communication with neurons as it facilitates the release of glial transmitters such as glutamate, adenosine triphosphate (ATP), and prostaglandin E2, which influence synaptic transmission and plasticity [74]. Astrocyte calcium signaling is disrupted in SMA, as shown by lower resting Ca^2+^ levels and an exaggerated rise in Ca^2+^ after ATP stimulation compared to WT astrocytes. This suggests that SMN deficiency affects calcium homeostasis, likely through altered purinergic receptor activity or calcium storage and release from the endoplasmic reticulum. Such disruptions may impair essential astrocytic functions like synaptogenesis, cell proliferation, and glutamate regulation, leading to impaired synaptic transmission and excitotoxicity. Notably, these calcium signaling defects appear exclusive to astrocytes, with lower MNs remaining unaffected [72].

#### 3.1.3. Evidence of Reactive Astrogliosis Preceding MN Degeneration

Increased GFAP expression and altered astrocyte morphology has been reported in SMA mice (Table 1). A hallmark pathological feature of SMA is the presence of glial bundles, which are thought to be astrocytic protrusions containing degenerated myelinated axons within neurilemmal tubes [42,75]. Both astrocytes and microglia lose SMN protein expression in SMA, leading to aberrant activation of glia and disruption of normal signaling. Optimal survival is achieved when SMN is highly expressed in both astrocytes and MNs [10,76]. Interestingly, selectively replacing SMN in astrocytes alone partially attenuated their pro-inflammatory profile, improved neuromuscular circuitry, and extended the life span of SMA model mice, suggesting that glia may contribute to the SMA disease phenotype [77].

It has been particularly highlighted that early GFAP upregulation is a marker of increased spinal astrocyte reactivity, preceding MN loss in severe SMA mouse models [23]. Furthermore, recent research has revealed elevated GFAP levels in the cerebrospinal fluid of patients with SMA [77,82]. It has been documented that media taken from SMA astrocytes can cause damage to both WT MNs and SMN-deficient MNs, underlining the disastrous effects that changed glial signaling has on SMA pathology [26,83].

Some groups have demonstrated that lower MN loss becomes detectable only in the final stages of SMA [77,84,85]. This suggests that MN death is a late event in the pathogenesis of SMA, preceded by the activation of glial cells, which may sustain and propagate the degenerative process [86]. Notably, research has identified that even prior to the degeneration of lower MNs, SMA astrocytes are pathologically activated, setting off harmful effects that further disrupt MN viability and cause nearby glial activation [10,30,87]. This pathological state is marked by increased microglial phagocytic activity, disrupted glutamate and serine metabolism, diminished neurotrophic support, elevated ROS production, and inflammatory markers such as GFAP, IL-6, and IL-1β and tumor necrosis factor alfa (TNF-α), as Table 1 shows.

Spinal astrocytes have been implicated in the pathogenesis of late-onset SMA, where early reductions in glutamate transport proteins, primarily excitatory amino acid transporter 1 (EAAT1), lead to glutamate excitotoxicity and subsequent MN loss (Table 1). Impaired or disrupted glutamate uptake by astrocytes can exacerbate hyperexcitation of N-methyl-D-aspartate and α-amino-3-hydroxy-5-methyl-4-isoxazole propionic acid (AMPA) receptors. Lower MNs are particularly vulnerable to AMPA-mediated glutamate toxicity, as calcium-permeable AMPA receptor subunits elevate intracellular calcium levels, ultimately driving MN death [88,89]. Ultimately, SMA astrocytes demonstrate impaired production of GDNF, dysregulation in microRNA expression profiles, and upregulation of nuclear factor kappa B (Table 1), all characteristics common to an inflammatory cellular stress response. These data show that astrocytes play an important role in the pathogenesis of SMA, and that they could represent an additional target for therapeutic intervention.

### 3.2. Microglia: Immune Surveillance and Synaptic Stripping

Microglial cells are highly branched resident macrophages with a non-neuronal origin from yolk sac erythromyeloid precursors [90]. Due to their continuous monitoring of the CNS and prompt phagocytic activity, microglia are considered the first immune response cells [91]. Morphologically, microglia are classified as ramified (resting) cells in search of signaling molecules, such as pathogen-associated molecular patterns and danger-associated molecular patterns which occur due to harmful stimuli, including cytokines, chemokines, purines, hormones, and neurotransmitters [91,92], after exposure to trauma, pathogens, stress, or the neurodegenerative process [28,93]. Upon such signal detection, microglia are activated and recruited in a process known as microgliosis, which can be detected with specific microglia markers, such as ionized calcium-binding adaptor molecule 1 (Iba1) [28,85,94] or the integrin CD11b+ [28]. Microglia also express the fractalkine receptor CX3CR1, CSF1R, surface glycoproteins F4/80 and CD68, and a pan-hematopoietic CD45 [91] marker. Activated microglia change their morphology from highly branched and ramified [95] to ameboid (phagocytic) and are able to remove cellular debris, misfolded proteins, and the cell remains [28,91,96]. At the site of pathogen or danger signal detection, activated microglia, besides morphological change, undergo metabolic changes that promote cytokine and chemokine secretion [28]. Microglial secretome recruits adaptive immune cells and plays a crucial, yet unclear, role in communication with neurons and neuroglia. While microglia and peripheral immune cells coordinate neuroinflammation, recent findings indicate that this process also manages tissue damage and repair simultaneously [93]. The adaptive immune response in neurodegenerative conditions involves the infiltration of mature T and B cells, which express unique antigen recognition receptors, allowing microglia to mount a targeted immune response during neuroinflammation [97]. However, it has been demonstrated that lymphocytic ablation does not change microglial activation and reactivity in SMA mice [28]. Also, in SMA experimental animals, spleen and thymus hypoplasia were detected, which can promote disease progression and neuroinflammation [98].

#### 3.2.1. Microglial Activation in SMA, Synaptic Stripping, and Its Implications

In SMA, microgliosis is considered to be excessive with a significant release of pro-inflammatory cytokines that further damage lower MNs and contribute to disease progression [28]. Current knowledge on microglia involvement in SMA is limited, but we know that microgliosis in the spinal cord has been detected post-mortem in patients with SMA [99,100].

Microglial activation [28,31,99,101,102,103,104], recruitment [28,30,85,94,99,101,104,105], and interaction with MNs [28,85,94,99,103] of the spinal cord has been detected in multiple studies using SMNΔ7 and some other SMA mice models (Table 2). Additionally, in an experimental severe SMA phenotype Taiwanese mouse model (*Smn^−/−^*, *SMN2^2TG/+^*), an increase in IL1b cytokine production in the spinal cord and brain was detected [106]. Even though microglial activation is evident *post-mortem* in the spinal cord of human samples [104] and SMA experimental animals (Table 2), in vitro studies using induced pluripotent stem cell (iPSC)-derived microglia from patients with SMA did not show microglial morphological changes, most likely because activation of microglia in SMA depends on the microenvironment and mutual communication with MNs and other glial cells [28]. However, these iPSC patient-derived microglia showed a reactive transcriptome profile, including upregulation in key genes responsible for cytokine signaling, innate and adaptive immune response, and toll-like receptors [28]. A study by Cervero et al. [99] emphasizes that SMA lower MNs trigger the formation of a pathological milieu that promotes glial activation and enhances physical interaction between glia and damaged MNs. Taken together, activated microglia in SMA can orchestrate lower MN survival [99].

However, studies have shown that the functionality of adult brain excitatory and inhibitory synaptic connections also require modulation by microglia [107]. During neurodevelopment, microglia are included in synaptic cleavage to ensure the strength of active neuronal pathways [108], while in adults they modulate learning and memory processes [96,109]. Numerous studies have shown microglial functions in synapse formation, elimination, and pruning in order to regulate synaptic function [91]. Namely, it has been reported that activated microglia can form physical contact with MNs and remove synapses from injured neurons in a process called “*synaptic stripping*”, which was first observed in 1968 [95,110]. Activated microglia have been observed to enwrap MN processes in experimental axotomy models [111]. However, during physiological CNS development and the establishment of neuronal circuits, the removal of inappropriate, supernumerary synapses requires a process dependent on C1q labeling [112]. Microglial tissue surveillance alongside synaptic stripping ensures tissue homeostasis in synaptic function and ensures synaptic plasticity and circuit refinement [113]. Enrolment of microglia in synaptic maturation and elimination has been considered necessary for normal brain development [114].

Interestingly, there have been studies that consider that MN death is microglia independent. Tarabal et al. [104] detected that MN loss in the ventral horn of the lumbar spinal cord happens before significant microglial reaction, indicating that microgliosis is not responsible for the induction of motor neuron death and is more likely a result of a secondary reaction from motor neuron dysfunction in SMNΔ7 mice. In addition, a study by Dachs et al. [105] showed that microglia do not promote MN destruction, as in the mouse model with a more severe SMA phenotype (FVB.Cg-Grm^7Tg(SMN2)89Ahmb^ Smn^1tm1Msd^/J) microgliosis was not present in the ventral horn of the lumbar spinal cord at the end of the animals life span of 6 days, when MN loss was maximal. Additionally, a study by Vukojicic et al. [94] proved that microglial depletion in SMA mice does not prevent motor neuron death in the spinal cord.

#### 3.2.2. Recent Findings on Complement Systems like C1q-Tagging Synapses for Microglial Phagocytosis

Synaptic elimination is a prerequisite for normal brain development and functional neuronal circuit establishment. In the developing CNS, C1q and C3 complement cascade proteins are abundantly expressed as they tag immature synapses for removal [89,112]. An initiating step of this complex cascade is coating synapses destined for removal, but also pathogens, dead cells, and debris in C1q complement opsonin [112,115]. In developing spinal cords, microglia are considered as the main source of C1q [28]. Synaptic coating with C1q activates downstream protein C3 convertase and induces the cleavage of C3 to C3a and C3b, while C3b binds to the targeted synapse [112,116,117]. C1q opsonin is also involved in microglia cytokine production as it reduces pro-inflammatory response [118].

Synaptic loss by the C1q complement system has been implicated in neurodegenerative diseases such as Alzheimer’s disease (AD) [119] and frontotemporal dementia [120]. Additionally, the C1q complement system has been abnormally activated in neurological conditions such as depression, with serological tests showing a significant increase in C1q levels in patients [121]. Interestingly, the upregulation of the C1q/C3-CR3 pathway was confirmed in an experimental depression mouse model, and treatment with neutralizing C1q antibody decreased microglia-mediated synaptic loss and improved behavioral outcomes [116].

Regarding SMA, Zhang et al. [122] detected dysregulation in genes involved in synaptic plasticity in lower MN and surrounding white matter from the spinal cord. Furthermore, synaptic pruning C1q complement mRNA and protein expression was increased alongside the mRNA upregulation of subunits C1qa, C1qb, and C1qc in lumbar MN of SMA mice. Vukojicic et al. [94] showed that in an SMA animal model, spinal cord synapses form normally prenatally but are eliminated at disease onset, reducing synaptic density. In SMA mice, C1q and C3 are upregulated in the spinal cord postnatally, activating the classical complement pathway in proprioceptive synapses. As inflammation markers were absent in mRNA, C1q likely originates locally near spinal cord MNs. Restoring SMN protein in proprioceptive neurons reduced C1q tagging of synapses. While lower MNs showed minimal C1q mRNA, microglia were identified as the primary C1q source. Anti-C1q antibody treatment in SMA mice inhibited synaptic coating and prevented synaptic loss. Increased C1q protein was also observed in Type 1 SMA patient spinal cord samples [94].

A transcriptomic analysis of SMA patient iPSC-derived microglia by Khayrullina et al. shows the upregulated expression of complement cascade-associated genes (C1qA–C, C5, C2) which indicates an increased phagocytic capacity of these cells [28]. It must be noted that the researchers did not detect the difference between the C3 expression of control and SMA-derived iPSC microglia, which were detected in SMA mice [28]. Injured neurons or astrocyte secretomes may influence C1q tagging and synaptic phagocytosis. Future co-culture studies could clarify the interactions between microglia, neurons, and other glial cells in C1q tagging and synaptic stripping. Blocking the C1q tagging system could protect synapses from degeneration, preserving neural connections in SMA. Targeting C1q in abnormal synapse elimination may offer a new therapeutic strategy to complement existing SMN protein restoration therapies, enhancing neuroprotection.

#### 3.2.3. Pro-Inflammatory Vs. Anti-Inflammatory (M1/M2) Microglial Responses

More than two decades ago, on the basis of in vitro testing, a binary model of microglia activation was adopted, in which microglia have two opposite states, an M1 pro-inflammatory/neurotoxic state (“classical activation”) and an M2 or anti-inflammatory/neuroprotective state (“alternative activation”) [99,110,123,124,125]. M1 microglia are mainly activated by pathogens or pro-inflammatory factors such as lipopolysaccharide, TNFα, and IFNγ and can be determined by surface receptors such as CD16, CD32, and CD86 membrane receptors. M1 secreted pro-inflammatory cytokines (IL1, IL6, IL12, IL17, IL18, IL23, TNFα and IFNγ) and inducible nitric oxide synthase (iNOS) can be considered as its markers [126]. The regenerative M2 phenotype is often labeled with a CD-206 marker and is activated with anti-inflammatory factors such as IL10 or IL4. The role of M2 microglia is obtaining homeostasis and secreting anti-inflammatory cytokines (IL-1 receptor antagonist (IL-1Ra), IL-4, TGFβ, IL-10, but also IL-4 and IL-13 receptor antagonist (IL-4Ra)), that are considered as M2 markers [28,99,126]. Determination of M1 or M2 phenotype dominance in tissue or cells is usually performed after labeling with pro- and anti-inflammatory markers and determination of M1/M2 ratio [126].

Studies have shown that microglia undergo phenotypic transition and promote neuroinflammation in neurodegenerative diseases, such as SMA [127]. In experimental studies, microglial polarization to M1/M2 was described in a few studies. A study by Cerveró et al. [99] detected a significant increase in M1 and a decrease in M2 microglia in the spinal cord of intermediate SMA phenotype mice (*Smn^2^*^B/−^). Interestingly, treatment with Sigma-1 receptor agonist PRE-084 corrected M1/M2 imbalance in the sense of more prominent M2 polarization but did not prevent lower MN loss. However, the beneficial effect of PRE-084 on gliosis could have the potential for further investigations into neurodegenerative disease. In SMNΔ7, the *SMN2 Smn^−/−^* SMA mice model, Khayrullina et al. [28] detected an increased number of CD86 positive reactive microglia in the lumbar part of spinal cords, which could be declared as M1 reactive phenotype cells. In another study, Karafoulidou et al. [101] detected an increased ratio of pro-inflammatory Iba1^+^iNOS^+^ cells and a decrease in anti-inflammatory Iba1^+^CD206^+^ microglia in the spinal cord and brainstem. These microglial phenotype changes were accompanied by prominent synaptic loss and a reduction in lymphoid organs and skeletal muscles.

Additionally, recent findings in single-cell sequencing have found a variety of microglial states depending on developmental stage, age, health, disease, and even brain regions [128,129,130]. For example, 57 microglia markers have been determined by multiplexed mass cytometry performed on human *post-mortem* donors [128]. However, these numerous microglial markers indicate microglial diversity and their ability for fast function changes, depending on the surrounding context, specifically the milieu and factors they are exposed to [99].

Current advanced SMA treatments, including gene therapies and SMN-modulating drugs, have improved patient outcomes by shifting the disease phenotype to milder forms. However, these therapies do not fully address all disease aspects, highlighting the innate immune system as a valuable therapeutic target. Modulating the CNS immune response, such as using microglia modulation drugs to promote an anti-inflammatory state, could complement existing therapies. The availability of new technologies like single-cell sequencing may enhance understanding and solutions for microglia polarization modulation.

#### 3.2.4. Oligodendrocytes and Schwann Cells: Myelination and Peripheral Nerve Involvement

Oligodendrocytes are a type of glia that produce axonal myelin sheath, a complex lipid structure that insulates neurons for an efficient action potential spread and also ensures axonal integrity [131,132]. Oligodendrocytes proliferate from oligodendrocyte progenitor cells (OPCs), also known as nerve/glial antigen 2 (NG2) cells, during neurodevelopment in a highly regulated molecular process that ensures appropriate OPC migration and differentiation [132]. In early embryonic life, OPCs are generated in the ventral neuroepithelium of the neural tube, while early postnatally they arrive from the dorsal spinal cord and hindbrain/telencephalon of the brain [133]. Oligodendrogenesis and myelin sheath production can also take place during adulthood and show plasticity that depends on environmental stimuli [134], which is also important in the treatment of SMA.

Demyelination, which happens as a consequence of oligodendrocyte disease, has been detected in multiple sclerosis and is accompanied by axonal degeneration [135]. However, oligodendrocyte disease can cause significant axonal loss in the brain without demyelination, as this has been detected in a mouse model with oligodendrocyte injury [136]. This indicates that oligodendroglia, aside from myelination, ensures neuronal survival and function by providing metabolic support [76,136]. Myelinated axons have high metabolic demands and rely on nodes of Ranvier for energy, making oligodendrocytes key energy sources. Studies indicate that oligodendrocytes produce various neurotrophic factors, including GDNF, BDNF, and IGF-1, which are altered in neurodegenerative diseases like multiple system atrophy [137].

Recent studies have shown that oligodendrocytes have an interdependent metabolic communication with neurons, showing abundant micromolecular communication through myelinic channels and periaxonal space [134]. Lee et al. [76] have shown the importance of the lactate transportation system in oligodendroglia functioning, as disruption of these lactate transporters can contribute to axonal degeneration and neuronal survival in vitro and the spinal cord. Interestingly, this was also confirmed in an ALS mouse model and in post-mortem tissue of patients with ALS [76], suggesting oligodendrocyte injury in neurodegenerative disease progression. Alongside loss of neurotrophic support, oligodendroglia could have a role in the activation of neuroinflammatory processes [8]. This is supported by the discovery of Bäumer et al. [138], who identified genetic alterations in spinal cord oligodendrocyte development in an SMA mouse model. In addition to communication with neurons, oligodendrocytes possess intensive communication with astrocytes as these types of cells form gap junctions which ensure homeostasis in potassium buffering and could also be important in axonal integrity maintenance, and they require more detailed studies [139], including ones in SMA.

Schwann cells are myelinating cells of the peripheral nervous system (PNS) that originate from the neural crest [133] and ensure precise communication with the CNS [140]. Schwann precursor cells differentiate into immature Schwan cells, which subsequently develop into either myelinating or non-myelinating Remak cells [141]. Myelinating Schwann cells have a canonical role in axonal ensheathment and myelination, a process performed during axonal sorting when they establish a one-to-one association with large axons designed for myelination. MNs and sensory axons are then wrapped with multiple concentric layers of myelin sheath. Birchmeier and Nave [142] reported on neuronal release of neuregulin-1, a differentiation factor that also promotes Schwann cells myelination. For the process of myelination, the activation of the PI3K/AKT/mTOR pathway and the ERK pathway is of the most importance [140]. Simon et al. [143] reported on the importance of a ciliary neurotrophic factor, highly expressed in myelinating Schwann cells surrounding axon terminals, and its importance in the sprouting response in the mouse SMA model.

Non-myelinating Schwann cells provide ensheathment without myelination to sensory axons involved in nociception and thermoreception [140,144]. Even though myelinating Schwann cells and oligodendrocytes myelinate neuronal axons in neurodevelopment, each Schwann cell is interconnected with only a short axonal section, while each multipolar oligodendrocyte can myelinate around axonal sections on numerous axons [139]. Schwann cells, the primary glial cells in the PNS, perform functions similar to oligodendrocytes, microglia, and astrocytes in the CNS. Unlike CNS glia, Schwann cells exhibit greater plasticity, retaining the ability to dedifferentiate or transdifferentiate even after becoming terminally differentiated. This plasticity of Schwan cells enables PNS to efficiently adapt to stress, disease, and injury, thereby providing robust resilience and a remarkable capacity for regeneration and repair [140]. Proteomic analysis of Schwann cells from SMA mice showed disturbance in the expression of genes involved in proliferation, survival, molecular transport, and ubiquitin pathways [145].

#### 3.2.5. Emerging Data on Oligodendrocytes’ Reduced Differentiation and Myelin Formation

OPCs are precursor cells of oligodendrocytes [132] which express a chondroitin sulfate proteoglycan characterized as a NG2. NG2 is a type of transmembrane protein, expressed in mammalian cells of neuronal and non-neuronal tissue, necessary for OPC proliferation during neurodevelopment and proper oligodendrocyte function [146]. OPCs express NG2 during adulthood, while myelin basic protein (MBP) is a marker of mature oligodendrocytes [147]. Only a few studies on oligodendrocyte differentiation and functionality in SMA are available to date, as Table 3 shows. A study by O’Meara et al. [27] did not reveal any differences in morphology, maturation, proliferation, migration, myelination, and cell death in an in vitro model of primary oligodendrocytes from a severe SMA mice model. The lack of oligodendrocyte differences in morphology, maturation, and myelination, between a severe SMA mice model (*Smn^−/−^*; *SMN2*) and WT mice was confirmed in in vivo studies by the same group as equivalent numbers of mature oligodendrocytes were found in corpus callosum, accompanied with normal myelination in the spinal cord [27]. However, a study by Ohuchi et al. [148] detected significantly lower NG2 and MBP expression levels in the spinal cord of SMNΔ7 mice and SMA-iPSC culture from patients with SMA, indicating the damage of oligodendrocyte lineage in SMA pathology. Furthermore, it was detected that oligodendrocyte proliferation disturbance, seen as the NG2 expression decrease, was induced with increased Notch1 signaling and inhibited Prox1 signaling, which develops before lower MN degeneration in SMNΔ7 mice [148,149]. Suppressed Notch1 signaling is considered of great importance in oligodendrocyte maturation [149] and its activation has previously been detected in spinal cord astrocytes lacking SMN protein as well as in the spinal cord astrocytes of SMNΔ7 mice [150]. Such discrepancies in oligodendrocyte pathology in SMA between these two studies could be explained by the type of chosen in vitro model. Namely, a study by Hauser et al. [151] emphasized that in vitro models of MN disease should rely more on iPSC-derived cultures and co-culture assays as suitable in vitro systems for obtaining disease-relevant results.

#### 3.2.6. Role of Schwann Cells in Peripheral Nerves and NMJ Maintenance

Schwann cells, as cells with high adaptation ability, have many canonical and noncanonical functions [140]. Regarding their canonical functions, one of the most important myelinating Schwann cell assignments is to provide support to long axonal fibers, as degeneration of these fibers is often considered the initial point of neurodegeneration [144]. Alongside structural support and protection, Schwann cells can monitor axonal metabolic status and provide metabolic support by insufficiently understood molecular pathways. Metabolism support to axons, as a function independent of myelination, involves proteins like MAG, proteolipid protein, and CNPase on the Schwann cells membrane, although precise axonal receptors remain elusive [140,152,153,154]. Substrates such as lactose, glucose, glycogen, and NAD+ as energy sources have been identified as compounds delivered to axons from Schwan cells [140]. Such metabolic status sensing, as a protective adaptation, has been reported after axonal injury in terms of a dynamic glycolytic shift modulated by mTORC1 [155]. Aside from ensuring axonal maintenance and regeneration, Schwann cells activated in a neurodegenerative environment can act as immunomodulators affecting both innate and adaptive responses [140].

The NMJ, formed between MN terminals and muscle fiber postsynaptic regions, is one of the most studied synapses [156]. Perisynaptic or terminal Schwann cells, a type of non-myelinating Schwann cell, envelop the presynaptic nerve terminal and postsynaptic muscle fiber, playing a crucial role in NMJ development [157]. They can be distinguished from myelinating Schwann cells by the simultaneous expression of S100 and NG2 markers [158]. Terminal Schwann cells in NMJ are crucial for synapse formation and their proper functioning and plasticity [159]. These cells are essential in NMJ trophic support but can modulate its synaptic communication by intracellular Ca^2+^ release via muscarinic and purinergic receptors, thereby modulating the release of neurotransmitters into the synapse [141]. Also, perisynaptic Schwann cells can release glutamate, nitric oxide, and prostaglandins for the neuromodulation of NMJ [141,160].

Following injury, terminal Schwann cells are essential for nerve terminal regeneration and repair [154] as these cells simultaneously regulate the development of both pre-and postsynaptic structures while undergoing intrinsic developmental maturation [161]. Experimental studies have shown the importance of terminal Schwann cells in synaptic transmission as their removal has been reported with more than 50% less synaptic transmission [140,162]. Terminal perisynaptic Schwann cells can extend long projections to adjacent NMJ after neuronal injury and allow axons to cross over for reinnervation [163,164]. Upon motor axon terminal degeneration, these cells secrete CXCL12α, which binds to the CXCR4 receptor on axon terminals and promotes NMJ regeneration [165]. Experimental NMJ denervation detected a decrease in muscarinic Ca^2+^ signaling which lasted 60 days post-injury, indicating glial cell memory of the injury [166]. In an intermediate SMA mouse model, Murray et al. [167] detected a reduction in the number of terminal Schwann cells per NMJ, but with their preserved ability to migrate and proliferate. Also, a significant reduction in terminal Schwann cells per NMJ area was reported in a pharmacologically induced SMA mouse model [168]. The loss of terminal Schwann cells could play a role in the decrease in structural plasticity which may impair compensatory reinnervation and affect the stability of NMJ [167]. Similar results on decreased terminal Schwann cell numbers in an SMAΔ7 mice model have been reported in a study by Lee et al. [169]. Ultrastructural analyses of NMJ detected vacuole-like inclusions in terminal Schwan cells and loss of identity cell marker S100 in several independent studies [170,171,172]. In NMJ with swollen mitochondria, but also in ones without observable mitochondrial damage, terminal Schwann cells are presented with inclusions such as vacuoles, and so these changes are considered to happen in early SMA and further promote NMJ damage [171,172]. Table 4 shows notable changes in Schwann cells.

#### 3.2.7. SMN Deficiency in Schwann Cells Impacts ECM Proteins and Axon Stability

An extracellular matrix (ECM) is a space between the cells built from a network of proteins, water, and saccharides that provides tissue support [177]. Proteins of the ECM provide structure and dynamics of the cytoskeleton in different cell types [178]. ECM of peripheral nerves is biochemically and structurally complex, and Schwann cells express various ECM receptors, making their interaction crucial for Schwann cell development and function. Notably, laminin signaling plays a key role in regulating Schwann cell proliferation, survival, differentiation, and morphogenesis [179]. Laminin as an ECM protein, when experimentally disrupted, diminishes the capacity of Schwann cells to project cytoplasmic extensions, which indicates that laminins play an important role in Schwann cells’ axon sorting and impact cytoskeleton organization [180]. In the spinal cords of pre-symptomatic [138,176] and post-symptomatic [176] SMA mice, alterations of ECM proteins gene expression, including laminin alpha 2 (LAMA2), have been found.

A study by Hunter et al. [174] demonstrated that Schwann cells lacking SMN protein show impaired maturation, myelination, interconnection with axons, and a defect in ECM composition in the peripheral nerves. Namely, in vivo experiments on two SMA mice models (Table 4) detected defects in the peripheral myelination process occurring postnatally, including low expression in essential myelin proteins and disturbances in axo-glial interactions at paranodes. Regarding ECM proteins, laminin expression was significantly decreased at the late-symptomatic time point. In vitro studies with primary Schwann cells from SMA mice showed abnormal differentiation and impaired production of key myelination proteins, which was reversed by SMN restoration. Co-culture experiments revealed that SMN-deficient Schwann cells could not efficiently myelinate healthy neurons, compromising neural stability. Schwann cell deficits were partially linked to LAMA2 protein expression, which decreased without a corresponding reduction in gene expression, suggesting post-translational processing involvement. Overall, the reduced SMN protein in Schwann cells disrupts ECM protein expression and function, affecting axon stability [174]. In a separate study by the same group [175], selective SMN protein restoration in Schwann cells corrected myelination abnormalities, improved neuromuscular function, and mitigated NMJ pathology in SMA mice but failed to influence lower MN soma loss. Finally, a study by Kong et al. [173] reported compromised motor axon radial expansion and deficient Schwann cell myelination in SMA mice, beginning during embryonic development and impairing motor axon function at birth. Postnatally, these poorly myelinated motor axons quickly degenerated. A reduced number of Schwann cells led to decreased axon–Schwann cell sorting and radial growth. Importantly, selective restoration of SMN protein in lower MNs significantly improved axonal growth and stability, while restoration in Schwann cells or muscle alone showed no benefits [173].

As both oligodendrocytes and Schwann cells have numerous important functions in normal MN functioning, future therapeutic options should include these cells for a more efficient myelination restoration and lower MN survival.

## 4. Neuroinflammation and Glial Cell Dysfunction in SMA Progression

“Neuroinflammation” refers to a complex set of molecular and cellular processes occurring within the CNS, characterized by the activation of glial cells and the infiltration of immune cells into nervous tissue [41]. Traditionally, neuroinflammation was viewed as a secondary response triggered by neuronal loss, limiting cellular damage and promoting regeneration within the CNS. However, recent research has revealed that neuroinflammation can also play a negative role in neurodegenerative diseases, including SMA. This shift in understanding highlights the complex interplay between glial cells and neurons, particularly how dysregulated inflammatory responses can contribute to neuronal apoptosis. Glial cell activation primarily initiates neuroinflammation in SMA. Gliosis has been observed in all forms of human SMA, occurring in regions of MN degeneration within the spinal cord and brainstem [8,41]. The relative contributions of changed astrocyte and microglia signaling to MN loss remain poorly characterized [181].

The quad-partite synapse is a complex, interdependent communication among astrocytes, microglia, and neurons within the CNS. Such dynamic interaction is based on highly responsive crosstalk of secreted factors, which allows glial cells and neurons to adapt and respond to changes in the cellular environment [23]. However, in diseases such as SMA these interactions become disrupted. Reactive microgliosis can recruit peripheral immune cells into the CNS, amplifying the inflammatory response. This environment creates a feedback loop that sustains neuroinflammation [182]. Microglia also adopt a pro-inflammatory phenotype in response to neuronal injury or stress, driven by NF-κB-mediated signaling. NF-κB regulates numerous pro-inflammatory genes, with sustained activation resulting in excessive ROS production, cytokine release, and neuronal apoptosis. Additionally, heightened NF-κB signaling increases microglial phagocytosis, which, when dysregulated, exacerbates neuronal damage. While this can be beneficial normally, excessive or uncontrolled phagocytosis leads to further neuronal injury. The release of pro-inflammatory cytokines further exacerbates glial activation [77,182]. Elevated levels of various pro-inflammatory cytokines are observed in patients with SMA. High serum concentrations of IFN-γ, IL-17a, and IL-22 suggest that the Th1/Th17 pathway is activated [183]. Recent research shows that activation of this pathway is related to disease activity in some neurodegenerative diseases which could mean that it also plays a role in SMA progression [25].

In SMA, aberrant Notch signaling has been implicated in the dysregulation of glial cell populations, particularly astrocytes, which significantly contribute to the disease’s pathophysiology. The Notch signaling pathway is an intercellular communication mechanism incorporated in many cellular processes. It is important for neural development and the maintenance of homeostasis. Studies indicate that astrocytes deficient in SMN protein exhibit upregulated expression of key components, such as the ligands Jagged1 and Delta1, along with increased levels of the Notch intracellular domain, a marker of pathway activation [150]. This dysregulated activation enhances astrogliogenesis, which may exacerbate neuroinflammatory and neurodegenerative processes in SMA [150,184]. Along with Notch signaling, NLR (NOD-like receptor) inflammasomes are also implicated as important in the pathophysiology of SMA. NLRP3, a cytoplasmic multimeric protein complex composite of an NLR, is upregulated in microglia from SMA mouse models. This leads to enhanced production of IL-1β and IL-18, which then continue to sustain the before-mentioned feedback loop [185].

Activation of the JAK2/STAT5 pathway also leads to the production of pro-inflammatory cytokines in microglia. Dysregulation of this pathway can polarize microglia toward an M1 phenotype. Studies have shown that increased activation of STAT3 correlates with elevated levels of inflammatory cytokines, indicating that persistent JAK/STAT signaling may contribute to chronic inflammation [186]. Additionally, complement proteins C1q and C3 are upregulated in response to neuronal injury in SMA. These proteins tag neurons for phagocytosis by microglia, which can lead to lower MN apoptosis [94]. This further shows the role of microglia in disease progression [187]. As stated, microglia are not the only cell types involved in SMA pathology. Altered astrocytes and neuronal signaling also influence it. Early in the disease, reduced expression of the EAAT1 glutamate receptor in astrocytes results in elevated glutamate levels within the spinal cord, which can lead to excitotoxicity and neuronal injury [79]. Furthermore, signaling pathways that regulate SMN expression, such as the MEK/ERK/Elk-1 pathway, have also been implicated in SMA. Studies suggest that inhibition of this pathway activates the AKT signaling cascade, leading to phosphorylation of CREB at Ser133. This activation promotes gene transcription in neuroprotection and cell survival, providing a potential therapeutic target [188].

These findings highlight the complex interactions between different cell types in SMA. The complex signaling pathways and their regulation challenge scientists working on finding potential therapeutic targets. Glial cells are key contributors to neuroinflammation, neuronal damage, and disease progression making them a great possible target for future drug development.

## 5. Potential New Drug Development Pathways Targeting Glia in SMA

### 5.1. Mechanistic Insights for Therapeutic Targeting

We have discussed the roles of microglia, astrocytes, and oligodendrocytes previously in the text, but it is important to highlight again the early involvement of glial cells in the pathogenesis of SMA. Several groups have shown the loss of lower MNs as an end-stage in the pathological cascade of SMA [84,85]. There are pre-synaptic defects that are influenced by the activation of glial cells due to their involvement in maintaining proper neurotransmission [86]. This is further proven by marked gliosis, which can be found in areas of MN degeneration throughout the spinal cord and brainstem [24] and has been shown to correlate with the loss of MNs [189]. The interplay between glial cells is critical for proper neural development [35], which implicates their dysfunction through all phenotypes of SMA, depending on the pathogenic variant present in patients. Likewise, it has been known for many years that the SMN protein plays a role in neurite outgrowth and neuromuscular maturation during development [190]. Still, a key development in the field is the advent and use of genetic therapy, as we are now entering into the uncharted territory of clinical phenotypes for patients, with the possibilities of supplementing currently available therapies with new targets, including glial cells [191].

Previous in vivo mice studies have pointed to a clear astrocytic dysfunction when the SMN protein is decreased [83]. Their regular homeostatic functions, such as supporting synaptic transmission, neuronal development, and beneficial factor secretion, are diminished in many neurodegenerative diseases, including SMA and MN disease [192]. In vitro studies have shown that astrocytes deficient in SMN exhibit significant alterations and functional impairments, including elevated GFAP, shortened processes (indicative of reactive astrocytosis), disrupted calcium homeostasis, decreased production of neurotrophins, and a reduced ability to support MN synapse formation [10,72]. Importantly, an in vivo mouse study has shown that spinal astrocyte dysfunction is particularly important in late-onset SMA, indicating the growing importance of targeting their function in future treatments, besides increasing SMN expression [79]. Numerous described reactive astrocyte signals and pathways are involved in homeostasis and cellular stress, such as STAT3, NFAT, and NF-κB, through which astrocytes enter an active phenotype that can be damaging if continuously activated [193]. The Notch signaling pathway has also been implicated as a mediator of astrocytic abnormalities in SMA models [183]. Possible targets to ameliorate astrogliosis include targeting their metabolism through mitochondrial modulation [194,195], targeting glutamate transporters EAAT1 and EAAT2 [196,197], or influencing cell-to-cell physical interactions via EPHB3 signaling [198].

SMN deficiency has been linked to a disruption in all aspects of protein homeostasis, such as RNA assembly, trafficking, translation, endocytosis, autophagy, cellular bioenergetics, and ubiquitin pathways [199]. The microglial function is intrinsically linked to all these functions, especially in their classical immunomodulatory polarization, considered as M1 and M2 [200,201]. Naturally, the reality of polarization is far more complex than the arbitrary two microglial “modes”, as novel research indicates [202,203]. Still, if we approach the system as binary, we can learn some mechanistic lessons about microglial activity and how to possibly modulate them in all diseases, as well as SMA [204]. The reaction of microglia and astrocytes share similar signaling pathways, such as NF-κB [205], but also TLR, STAT, and Notch pathways [206,207,208]. NF-κB has been implicated in both ALS and SMA, as the deficiency of SMN protein in microglia promotes oxidative stress and activates the pathway [118]. Further possible targets based on the pathogenic mechanisms are acting on the complement system, as the classical complement pathway is known to mediate microglia-dependent remodeling in development and SMA [93]. The key initiator is known as C1q, and there are in vivo studies that show the inhibition of this initiation leads to a rescue of synapses in SMA mice [93] and plays a key role in maintaining functional motor circuits [119]. There are already multiple approved complement blockers in use today for a wide range of disorders, from systemic disorders like atypical hemolytic syndrome, anti-neutrophil cytoplasmic antibody vasculitis, to neurological disorders like myasthenia gravis (MG) and neuromyelitis optica [209], and could present an opportunity for use in SMA as well to supplement genetic therapies.

Looking at the mechanisms of glial activation, another possible target for action is the sigma-1 receptors (Sig-1Rs), which are transmembrane proteins in the endoplasmic reticulum [210]. They appear to be key in neuronal survival and function due to a modulation of calcium homeostasis and glutamate activities, which influence reactive gliosis [211]. They are highly expressed in both astrocytes [212] and microglia [213], and targeting Sif-1Rs with ligands appears to induce a favorable phenotype for tissue repair and regeneration [214,215]. Several studies have shown that targeting Sig-1Rs in ALS leads to neuroprotection, especially in SOD1 variants [216,217]; however, there is a lack of studies focusing on SMA.

It should be said that targeting glial cells and neuroinflammation in neurodegenerative diseases has not yet given the expected results [218]. Studies in the field of AD, Parkinson’s disease, and ALS showed promise in the preclinical trials, but have not yet produced the desired results [219]. Novel research in the AD field shows a varied microglial activity depending on the stage of disease, which could hold answers on the complexity of targeting glial polarization [220]. As an example, depleting homeostatic microglia in early stages leads to a reduction in plaque numbers and neuritic dystrophy, while in the later stages the activated microglia reshape the amyloid plaques in a protective manner [221]. Therefore, timing seems to be a key factor in choosing to modulate neuroinflammation, as well as the selectiveness of agents used. In general, considering the benefits of inflammation in maintaining homeostasis, we must be careful not to overly suppress homeostatic glial activity and interfere with their function [222].

### 5.2. Emerging Therapeutic Strategies

Gene therapies targeting astrocytes and microglia have emerged as promising strategies to restore SMN protein function, particularly in the context of neurodegenerative diseases such as SMA [223]. This approach focuses on leveraging the unique roles of these glial cells in neuroinflammation and neuronal support. Studies have shown that conditioned media from SMA astrocytes can negatively impact MNs, leading to shorter neurites and decreased expression of MN-specific markers. This suggests that factors secreted by dysfunctional astrocytes contribute to MN loss. Regarding microglia, the interplay between them and astrocytes could contribute to neuroinflammatory processes observed in SMA, thus abnormal synaptic pruning by microglia could lead to further MN deafferentation, worsening the disease state [24,224]. Recent key improvements with gene delivery systems targeted at astrocytes have been developed using adeno-associated virus vectors. These vectors can be engineered to express therapeutic genes specifically in astrocytes, potentially enhancing SMN protein levels. Of note, recent studies have reported that astrocyte-targeted delivery of IL-2 can modulate inflammatory responses effectively without affecting peripheral immunity [225]. Microglial gene therapy targets the modulation of microglia activity with the aim of driving their phenotype toward protection. For instance, intervention strategies in microglial signaling pathways may improve their homeostatic restoration capability in neurodegenerative contexts. Targeting selective receptors on microglia has also been associated with reduced chronic inflammation in diseases such as SMA [90,226].

Furthermore, there is emerging evidence that blocking microglial-mediated synapse removal through monoclonal antibodies against complement proteins, in particular C1q, may represent a new therapeutic strategy to ameliorate neurodegenerative processes [227]. C1q is a central component of the complement cascade that tags synapses for elimination by microglia. During neurodevelopment and in pathological conditions, C1q binds to synaptic elements, marking them for phagocytosis. In turn, microglia express complements, protein-recognizing receptors that promote the engulfment of tagged synapses. This process is of the utmost importance for maintaining synaptic homeostasis; however, it can lead to excessive synapse loss in diseases such as schizophrenia and AD when dysregulated [228,229]. Microglia use a variety of signals to identify and prune synapses. The balance between these signals determines which synapses are preserved or eliminated [230,231]. In pathological states, an imbalance between the signals may cause inappropriate synapse removal, leading to cognitive deficits and other neurological symptoms [229]. Monoclonal antibodies, which target C1q, can block its interaction with microglial receptors, and consequently prevent the inappropriate tagging of synapses for removal. This therapeutic approach aims to preserve synaptic integrity and function by preventing complement-mediated phagocytosis, responsible for synaptic loss. Published literature demonstrates that blocking microglial phagocytosis can prevent the loss of inhibitory synapses, which is crucial for maintaining excitatory–inhibitory balance in neural circuits [228,232]. Data reported by preclinical studies indicate that inhibiting microglial activity through complement blockades can modulate synaptic connectivity positively. For instance, the interference of microglial phagocytosis in neuropathic pain models prevented the loss of specific spinal synapses, which impeded neuroinflammation-induced synaptic alterations [232].

Anti-inflammatory and neuroprotective agents, such as monocyte chemoattractant protein-1 (MCP-1) analogs and microRNA inhibitors, have been tested with promising results in terms of the induction of neuroprotection through the regulation of glial responses and cytokine-induced neuroinflammation [233]. microRNA miR-146a is known to regulate the inflammatory response by targeting two major components in the NF-κB signaling pathway, namely interleukin-1 receptor-associated kinase 1 and tumor necrosis factor receptor-associated factor 6 [234]. By targeting these, miR-146a suppresses the activation of NF-κB, which thus leads to reduced production of pro-inflammatory cytokines such as IL-6 and TNF-α [235,236]. miR-146a has been proven to suppress inflammatory responses in many models, including neurodegenerative disorders like AD and multiple sclerosis. Its overexpression may cause a decrease in neuroinflammatory markers, thus promoting a healthier neuronal environment [236,237]. MCP-1 is involved in recruiting immune cells to sites of inflammation. Analogous compounds can be designed to either mimic or inhibit MCP-1 activity, potentially altering glial cell responses. Analogous compounds of MCP-1 may modulate microglia and astrocyte responses, directing them from a pro-inflammatory state toward an anti-inflammatory one. Such modulation has the potential to prevent neuroinflammation with related neuronal damages associated with chronic inflammatory conditions [238]. Both miR-146a and MCP-1 analogs have therapeutic potential in various neurodegenerative disorders. miR-146a can downregulate key inflammatory pathways, making it a candidate for therapies for conditions characterized by excessive inflammation, such as AD and ALS [235,236]. MCP-1 analogs could be utilized in conditions where modulation of immune cell recruitment is beneficial, potentially reducing the detrimental effects of chronic inflammation on neuronal health [239]. These agents could thus facilitate the development of new therapeutic approaches that would not only reduce symptoms but might also affect basic inflammatory mechanisms. Future studies will be important to establish optimal dosage, mode of delivery, and long-term impact on neuronal viability.

## 6. Challenges and Future Directions

### 6.1. Beyond Restoring SMN Protein Function in Neurons

Available therapies are changing the SMA landscape, with apparent disease-modifying effects. Meta-analyses show that gene therapy benefits patient survival and improves motor function, with the most effectiveness being in onasemnogene abeparvovec and risdiplam for SMA type 1 and 2 [240]. The effects of restoring SMN function extend beyond neurons, as research shows a significant effect of therapy on neuroinflammation, myelination defects, and neuromuscular function [77,173]. Thus far, most published studies are performed in patients on nusinersen, and point to an immunomodulatory effect by reducing the cerebrospinal fluid levels of several pro-inflammatory mediators and an increase in trophic factors, even though the mechanisms are not yet clear [241,242,243]. On the other hand, it seems that adaptive immunity is not affected by nusinersen treatment, indicating that this immunomodulatory effect may be due to an effect on microglia [244]. To the best of our knowledge, no published studies focus on glial cells and the therapeutic effects of risdiplam or onasemnogene abeparvovec. It is now essential to carry out further preclinical and clinical research to elucidate the mechanisms, which are the effector cells and the rate at which the SMA protein function recovers in glial cells. An additional problem brought about by SMN protein modulation is that not all patients are responders, and that there are other mechanisms that affect disease progression aside from lacking SMN protein expression, creating a need for SMN-independent treatments [245].

Patients treated with disease-modifying genetic therapy will invariably lead to new clinical phenotypes and thus require novel supportive therapy that can be repurposed from other indications. Glutamatergic neurotoxicity driven by astrocytes is increasingly being implicated in the pathogenesis of SMA, especially with increased patient survivability [79]. A possible treatment for this pathogenic mechanism is riluzole, a glutamate inhibitor routinely used in ALS [246]. Its primary mechanism involves inhibiting pathological glutamatergic transmission by blocking sodium channels, thereby reducing excitotoxicity, which is a process implicated in SMA neuronal damage [247]. Neurons with lowered SMN protein expression could especially be susceptible to this pathogenic mechanism, as it includes oxidative stress and altered mitochondrial dynamics [248], even though there are opposing results on whether this is relevant in SMA [87,249]. There is limited in vitro evidence that riluzole could ameliorate the damage caused by the diminished SMN function [250], and even though a phase 1 trial had promising results [251], no further studies were performed in the field. Another ALS therapeutic agent, edaravone, is also a candidate for repurposed use in SMA [252], with its mechanism of action focused on neutralizing reactive oxygen and nitrogen species and reducing oxidative stress [253]. Aside from riluzole and edaravone, there are numerous drugs with repurposing potential that focus on neuroprotection, muscle strength, NMJ function, and cell death pathways [254]. Neuroinflammation remains a promising target, especially with novel treatments like complement blockers [255], although further research into the mechanics of the disease is needed.

Microglia have been considered a valuable therapeutic target in neurodegenerative disease as enhancing their phagocytic activity, suppressing microglial-driven neuroinflammation, and encouraging the transition to a protective phenotype are observed as promising therapeutic approaches [256]. Advancements in SMA treatment have markedly improved patient outcomes but do not fully address all aspects of the condition. Additional strategies, such as modulating the CNS’s immune response with microglia-targeted drugs that promote an anti-inflammatory phenotype [98], could be beneficial alongside current treatments. Future studies including advanced technologies like single-cell sequencing will provide valuable insights and innovative solutions for regulating microglial polarization. Inhibiting the C1q tagging pathway may safeguard synapses from degeneration and loss, thereby preserving neural connections in SMA. Synaptic loss regulated by C1q tagging has been experimentally restored with the application of an anti-C1q antibody [93]. It presents a promising therapeutic target, since it was found post-mortem that C1q protein expression and complement cascade-associated genes [92] have been upregulated in the spinal cord of patients with SMA [93]. Addressing abnormal synapse elimination could represent a novel therapeutic approach, offering a more comprehensive neuroprotective strategy.

Recovery of astrocytic functional capabilities impaired in SMA, including GDNF production, microRNA profile regulation, and suppression of astrocyte-regulated inflammation, can be considered as potential therapeutic targets in future preclinical investigations [10,26,77]. Given the critical roles of both oligodendrocytes and Schwann cells in maintaining normal lower MN function, future therapeutic strategies should also incorporate approaches targeting these cells to enhance myelination restoration and improve MN survival more effectively. Furthermore, future preclinical studies on the restoration of terminal Schwann cells could have a significant impact on SMA treatment as these cells have high importance in the development of both pre- and postsynaptic structures [160], as shown by their reduction per NMJ which has been reported in different SMA models [166,167,168].

### 6.2. PNS Involvement and Its Therapeutic Targeting

Addressing PNS involvement, particularly regarding Schwann cells and NMJs, is crucial for developing effective treatments for multiple neuromuscular disorders. The literature indicates their key role in disease progression and as potential points of intervention. Of utmost importance, Schwann cells myelinate peripheral nerves, support nerve regeneration, and maintain neuronal health. They also play a critical role in the process of nerve repair following injury through the promotion of axonal regrowth and remyelination [143,257]. On the other hand, various diseases, including diabetic neuropathy and CMT disease, involve impaired Schwann cell function leading to abnormal nerve maintenance and faulty nerve repair that results in chronic pain [258]. Approaches to the targeting of Schwann cells for therapy may be either gene therapy or regenerative factors. Gene therapy includes the use of adeno-associated viruses that deliver genes that enhance Schwann cell function or promote myelination in preclinical models, which restores function to the diseased or injured nerves. Regarding regenerative factors, the addition of factors like GDNF and neuregulins may be utilized to augment the activity of Schwann cells in support of nerve regeneration [259,260].

Alterations at the NMJ result in muscle weakness and atrophy, characteristics of neuromuscular diseases such as MG and SMA [261,262]. Recent efforts have been put into various strategies targeting NMJs for therapeutic benefits, namely through molecular intervention and monoclonal antibodies. Relating to the first one, agents facilitating the clustering of AChRs or the stabilization of NMJ structure are capable of improving synaptic transmission. For instance, AAV-mediated gene therapy has been adopted for the overexpression of proteins including MuSK, an integral component of NMJ integrity [260,263]. With respect to the second one, complement protein targeting at the NMJ can deter autoimmune responses in MG. One single-chain antibody, targeted against AChRs, has proven effective in reducing complement deposition and associated muscle weakness in animal models [264]. Ongoing clinical trials are testing agents that enhance the NMJ function; among them, agonist antibodies which stimulate the NMJ associated molecules, delaying the degeneration and drug repositioning, with currently available drugs such as amifampridine being investigated for its efficacy in enhancing this [260].

Targeting Schwann cells and NMJs thus offers a multilayered platform for the treatment of PNS disorders, which leverages gene therapy, regenerative factors, and immunomodulatory strategies. Researchers aim to restore normal function and improve outcomes for patients with neuromuscular diseases. Their continued exploration will thus be critical in developing effective therapies aimed at symptomatic relief and addressing the underlying pathology.

### 6.3. Potential Role of Adaptive Immunity and Its Therapeutic Exploration

Adaptive immunity plays a crucial role in protecting against infectious diseases and malignancies, while it uniquely involves a diverse lymphocyte repertoire through T cells and B cells that respond in a highly specific manner against a variety of pathogens and tissue damage. Recent research highlights how adaptive immunity could play a role in therapeutic situations, especially in the treatment of cancer and neurodegenerative diseases [265]. Another salient characteristic is lymphocyte activation: antigen-specific recognition through T cells and B cells leads to the initiation of intracellular signaling cascades that, in turn, lead to immune cell proliferation and differentiation into effector and memory cells. These memory cells allow for a more rapid response following re-exposure to the same antigen, amplifying protective immunity [266]. Secondly, cytokine signaling, where communication between lymphocytes occurs through soluble proteins such as cytokines and chemokines. These signals modulate the functional state of immune cells, influencing their activity and interactions within the immune network [266,267].

Published literature indicates that adaptive immunity has been identified as greatly contributing to the overall efficacy of targeted cancer therapies. Monoclonal antibodies used for the treatment of tumors, for instance, have been reported to activate the host immune system, which assists in tumor regression by showing immune-mediated cytotoxic effects [268].

Clinical trials have demonstrated that patients receiving treatments such as trastuzumab display higher levels of CD8 T cells and natural killer cells, associated with improved clinical outcomes [266,267]. Furthermore, there is a growing interest in combining immunotherapies with targeted therapies since this would not only enhance immune responses but also increase tumor control. It is essential to know the interaction of these various targeted therapies with the immune system to optimize such combinatorial strategies [266,267]. Lastly, adaptive immunity is also being explored in treating neurodegenerative diseases such as AD. The approval of aducanumab, a monoclonal antibody targeting amyloid β aggregates, exemplifies this approach. Although its efficacy remains controversial, ongoing research focuses on utilizing adaptive immune responses to restore immune balance in AD [266,267]. Investigating the functions of T cells, B cells, and cytokine signaling in SMA may reveal innovative methods to adjust immune responses, lessen inflammation, and improve the effectiveness of gene or antisense therapies. This developing area highlights the necessity for focused research on the immune system’s role in SMA, aiming to enhance treatment strategies and patient outcomes.

If the previous decades of research were focused on the neuron, recent years have brought about the years of glia. There has been a steady expansion of research focusing on the supportive cells in the central nervous system, with rapid discoveries in novel functions happening on a monthly basis [269]. Even though every disease is a story of its own, some lessons can be learned and applied in more than one condition, as can be seen in Alzheimer’s or Parkinson’s disease [91]. Even though a detailed review of the mechanisms in each disease is beyond the scope of this review, we can highlight some more common and rarer conditions and glial involvement. One lesson we can see in Alzheimer’s or Parkinson’s disease is the dual nature of microglial function. For example, we now have significant evidence that microglia promote the uptake and degradation of beta-amyloid and tau protein, which can be considered useful in some disease phases, but has conversely also been linked with pathological propagation and worsening [270]. Similar can be seen in Parkinson’s disease, as microglia has a clear role in the phagocytosis of α-synuclein, which can in turn cause a vicious neuroinflammatory cycle that is difficult to break [271,272].

Looking at some rarer diseases and syndromes, in Alexander’s disease there is a clear role for astrocytes due to the pathological variants in the GFAP gene. The gene encodes a protein that is a major intermediate filament of astrocytes, and the GFAP has been linked to many neurodegenerative diseases as a potential biomarker [273]. As such, Alexander disease is an example of a primary gliopathy, or in this case, an astrocytopathy, which leads to leukodystrophy [274,275]. Mutations in other glial genes, such as the MECP2 gene [276], have been linked with Rett syndrome, and in the FMR1 gene for fragile X syndrome [277]. Pathological variants cause numerous alterations that are not only present in the brain, but much of the function is focused on the maturation and function of all glial cells, including oligodendrocytes [278,279]. Glial involvement is also seen in chromosomal disorders like Down syndrome, with an altered function of all glial cells along with defective neurogenesis [280,281]. Furthermore, genetic heterogenous autoimmune disorders, like Aicardi–Goutières syndrome, are associated with increased interferon-α production, which is a key cytokine of astrocytes in the central nervous system [282]. Looking away from genetic disorders toward metabolic disorders, glial cells have a key role in the protection of neurons from alcohol and other toxic substances [283,284]. Chronic exposure to alcohol can disrupt their function, and in turn, cause significant apoptosis of neurons mediated by microglial cells [285]. Overall, we must have an open mind and look at the wide picture when examining the pathophysiology involving glial cells, as many beneficial functions they perform can quickly turn towards harm. Treatment involving glial cells must take into account the component of intervention timing, and this is something that will need to be researched as we enter a new phase of SMA research and treatment.

While much progress has been made, there are challenges ahead to fully determine the mechanisms whereby active immunity plays its role in disease and therapy. Studies will need to be directed at providing this understanding and identifying appropriate agents that can modulate adaptive immune responses without toxicity or serious adverse events. Once those are fully understood, a real personalized medicine strategy utilizing adaptive immunity can be developed leading to improved health outcomes [267,286,287].

## 7. Conclusions

In conclusion, while current SMN-targeted therapies have transformed the landscape of SMA management, they fail to fully address the disease’s non-cell-autonomous mechanisms, particularly the contributions of glial cell dysfunction. Emerging evidence highlights the critical roles of astrocytes, microglia, and oligodendrocytes in SMA progression through disrupted neurotrophic support, synaptic instability, and heightened neuroinflammation. By targeting these glial cell-mediated pathways, future therapeutic strategies could complement existing approaches, providing a more holistic treatment framework. Advancing our understanding of glial biology in SMA is imperative to unlocking novel avenues for improving patient outcomes and quality of life.

## Figures and Tables

**Table 1:** Selected in vivo and in vitro studies on the role of astrocytes in SMA.

In Vivo	
Model/Genotype	Notable Changes in Astrocyte Morphology and Function
SMNΔ7mice [10]	SMA astrocytes in spinal cord exhibit distinct morphological changes alongside upregulated GFAP and Nestin expression [10].
SMNΔ7 and the less severe *Smn^2^*^B/−^model, SMNΔ7 mice treated with scAAV-SMN^gfap^ [77]	- Restoring SMN expression in astrocytes extends lifespan and markedly enhances motor function, while partially recovering deficits in NMJs and proprioceptive synapses [77]. - The presence of astrogliosis with GFAP staining was progressively more widespread in the lumbar spinal cords from end-stage SMA mice [77]. - Progressive increase in the expression levels of IL-1β, IL-6, and TNF-α which were partially normalized in treated mice [77].
SMNΔ7mice [26]	miR-146a was markedly upregulated in spinal cords [26].
SMNΔ7 mice (Hung model) [78]	- Astrocyte-specific proteins, Kir4.1 and EAAT1, were decreased before the first signs of spinal MN loss [78]. - Elevated extracellular glutamate levels and disrupted potassium regulation were associated with reduced EAAT1 and Kir4.1 [78].
SMNΔ7 mice (Hung model) [79]	- Increased GFAP levels as a marker in SMA mice before the loss of spinal MNs. - Arundic acid in SMA mice enhanced EAAT1 expression and reduced glutamate levels, improving the progression of SMA by preventing MN loss and mitigating muscle fiber degeneration [79]. - AA also slowed down the denervation of NMJs, although it did not provide full protection [79].
SMNΔ7 mice [80]	- Impaired glutamate–glutamine metabolism in the brain and spinal cord of SMA mice [80]. - Supplementation with D-serine enhances motor function to a moderate extent in SMA mice [80].
**In Vitro**	
**Model/Genotype**	**Notable Changes in Astrocytes Morphology and Function**
iPSC-derived SMA astrocytes from a human SMA patient [10]	- SMA astrocytes exhibit distinct morphological changes alongside upregulated GFAP and Nestin expression [10]. - Elevated basal calcium levels and impaired ATP-stimulated responses [10]. - ERK1/2 activation and decreased GDNF secretion [10].
primary cultures of pure MN and astrocytes from WT and SMNΔ7mice along with their mixed and matched co-cultures [72]	- SMN deficiency in lower MNs impairs synapse formation and function [72]. - SMN deficiency in astrocytes disrupts calcium homeostasis and alters Ephrin B2 expression [72]. - SMN deficiency affects synapse formation and function in co-cultures of MN and astrocytes [72].
iPSC-derived SMA astrocytes—from a human SMA patient/iPSC from a health control patient [26]	- miR-146a was identified as the sole miRNA that was both highly and significantly upregulated in SMA astrocytes and their secreted exosomes [26]. - Increased miR-146a levels caused spinal cord MN loss, whereas miR 146a inhibition prevented SMA astrocyte-induced MN loss [26]. - A significant increase in the expression of NfκB and GATA6 [26]. - Decreased GDNF production [26]. - GDNF re-expression did not decrease astrocyte reactivity [26].
iPSC-derived SMA astrocytes—from a human SMA patient/iPSC from a health control patient [30]	- SMA astrocytes exhibit aberrant activation marked by increased pro-inflammatory signaling (GFAP, IL6, IL1β) and reduced neurotrophic support, driving toxicity to MNs [30]. - GATA6 drives astrocyte inflammation and NFκB signaling, with its knockdown reducing inflammatory markers in SMA astrocytes and improving spinal MN survival [30]. - SMA astrocyte-conditioned media activated SMA microglia, emphasizing an astrocyte-first mechanism in the neuroinflammatory cascade [30].
MNs and spinal astrocytes transfected with SMN siRNA from WT mice [78]	- SMN-deficient astrocytes showed increased GFAP expression and ROS production reduced Kir4.1 current density depolarized resting membrane potentials [78]. - Hyperexcitability and increased calcium spiking frequency in cultured spinal MNs [78].
iPSC-derived spinal MNs and SMA astrocytes—from a human SMA patient/iPSC from a health control patient [81]	- SMA astrocytes exhibited defective glutamate uptake due to EAAT1 impairment [81]. - Selective EAAT1 inhibition in healthy co-cultures replicates the reduced MN activity observed with SMA astrocytes [81]. - Increased SMN protein levels in SMA astrocytes partially restore EAAT1 expression and function [81].
MNs and spinal astrocytes transfected with SMN siRNA from WT mice [79]	SMN-deficient astrocytes display reduced EAAT1 protein levels, resulting in elevated glutamate concentrations [79].

ATP, adenosine triphosphate; EAAT1, excitatory amino acid transporter 1; Erk1/2, extracellular signal-regulated kinases 1 and 2; GATA6, GATA-binding protein 6; GFAP, glial fibrillary acidic protein; GNDF, glial cell line-derived neurotrophic factor; iPSC, induced pluripotent stem cell; Kir4, inwardly rectifying potassium channel 4.1; miRNAs, microRNAs; MN, motor neuron; NfκB, nuclear factor kappa-light-chain-enhancer of activated B cells; NMJ, neuromuscular junction; TNF-α, tumor necrosis factor alfa; ROS, reactive oxygen species; SMA, spinal muscular atrophy; SMN, survival motor neuron; WT, wild-type.

**Table 2:** Selected in vivo and in vitro studies on the role of microglia in SMA.

In Vivo	
Model/Genotype	Notable Changes in Microglia Morphology and Function
SMNΔ7 mice [101]	- Higher ramification index of Iba1^+^ cells and increased numbers of pro-inflammatory Iba1^+^iNOS^+^ microglia in the spinal cord and brainstem [101]. - Systemic administration of LPS led to a significant elevation in the number of microglia exhibiting the reactive phenotype in the spinal cord and brainstem [101].
SMNΔ7 mice [28]	- Microglial reactive morphology is characterized by enlarged soma, shortened and less complex processes, and increased surface expression of the activation marker CD86 in the spinal cord [28]. - No evidence of increased microglial proliferation in the spinal cord or infiltration of peripheral immune cells at the end stage of the disease [28].
SMNΔ7 mice [31]	- The number of activated microglia in the spinal cord was elevated in SMNΔ7 mice compared to WT mice, but this increase was suppressed following administration of an antisense oligonucleotide (SMN-ASO), which elevates SMN protein levels [31]. - A significantly higher number of 8-OHdG-positive cells (a marker of oxidative stress-induced DNA damage) among Iba1^+^ microglia in the spinal cord of the SMA mice compared to WT mice [31]. - ASO treatment decreased the expression of oxidative stress markers in the spinal microglia within the SMA model mice [31].
SMNΔ7, C1q-, SMNΔ7/C1q-mice [94]	- Microglia are the exclusive source of C1q production in the spinal cord of both WT and SMA mice [94]. - In SMA mice, microglia exhibit abnormal upregulation of C1q protein which is associated with the dysfunction of vulnerable synapses on lower MNs marked by C1q deposition [94]. - C1q deposition on synapses initiates activation of the classical complement cascade, resulting in their elimination through microglia, likely via complement receptor 3 [94]. - Pharmacological inhibition of C1q or microglia depletion provides therapeutic benefits in SMA mice, including improvements in the righting reflex [94].
SMNΔ7 and *Smn^2B/−^* mice [99]	- Prominent microgliosis around MNs in the ventral horn of lumbar spinal cord in *Smn^2B/−^* mice [99]. - *Smn^2B/−^* mice compared to WT mice exhibited a striking increase in harmful M1 microglia (Mac-2-positive) and a significant decrease in beneficial M2 microglia (CD206-positive) within the spinal cord [99]. - Sig1R agonist (PRE-084) decreased the density of M1 microglia and increased the number of M2 immunolabeled microglia in the spinal cord of *Smn^2B/−^* mice, although it fails to prevent MN loss [99].
TDP-43^A315T^ x SMN, TDP-43^A315T^, SMN and WT mice [103]	- Increase in microglial activation in the spinal cords of TDP-43^A315T^ mice compared to WT [103]. - Significant reduction in microglial activation in TDP-43^A315T^ x SMN mice relative to TDP-43^A315T^ mice suggests that the reduced activation of microglia due to SMN overexpression may contribute to the protection of motor neurons [103].
SMNΔ7 mice [104]	- After MN death occurs at P0–4, at later time points, P7–8 to P14–15 microglial activation occurs in the ventral horn of the lumbar spinal cord [104]. - Increased density of microglial cells in the lumbar spinal cord of SMNΔ7 mice compared to WT mice is accompanied by the significant increase in Iba1^+^/CD68^+^ microglia [104]. - Ultrastructural analyses show that microglia actively phagocytose nearby degenerating presynaptic terminals, highlighting their involvement in the clearance of damaged synaptic components in the spinal cord [104].
SMN^−/−^ and *SMN2*^+/+^ Mice [105]	- No significant differences in the number of Iba1^+^ microglial cells in the ventral horn of the lumbar spinal cord between WT and SMA mice, indicating the lack of prominent microgliosis in the spinal cord [105]. - In the pathogenesis of the most severe SMA phenotypes, unlike the SMNΔ7 model, neuroinflammation may not play a crucial role [105].
SMNΔ7 mice [85]	- Microglial density in the spinal cord of SMNΔ7 mice increases by 34% at P4 and 65% at P14 compared to control mice [85]. - Microglia in the lumbar spinal cord of SMNΔ7 mice are activated by their larger cell bodies, intense Iba1 immunoreactivity, and shorter, thicker processes [85]. - Association of microglia with MNs is observed in the lumbar spinal cord of SMNΔ7 mice [85].
**In Vitro**	
**Model/Genotype**	**Notable Changes in Microglia Morphology and Function**
iPSC-derived SMA microglia—from a human SMA patient/iPSC from a health control patient [28]	- SMA microglia had significantly fewer processes compared to the control [28]. - Control and SMA microglia exhibited similar cell soma sizes, which contrasts with the findings observed in SMA mice and suggests that the microenvironment likely plays a role in shaping some of the morphological characteristics of SMA microglia [28]. - iPSC-derived SMA microglia displayed a reactive transcriptomic profile, characterized by increased cell migration and enhanced phagocytic activity [91]. - The secretome of SMA microglia induces changes in the morphology and electrophysiological properties of MNs [28].
iPSC-derived microglia—from a human SMA patient and control—exposed to SMA iPSC astrocyte-conditioned media (ACM) [30]	SMA astrocytes contribute to the microglial phagocytosis observed in SMA, with this process being significantly reduced following lentiviral-mediated knockdown of GATA6 [30].
siRNA SMN-depleted RAW264.7 macrophage cells [31]	Depletion of SMN protein in RAW264.7 cells enhanced oxidative stress and elevated the inflammatory response by phosphorylation of NFκB and JNK [31].
BV2 microglia cells [102]	- RNA interference-mediated depletion of SMN enhances IL-1β-induced activation of IKK, which subsequently boosts the production of inflammatory mediators such as TNF-α and NOin microglia lacking SMN [102]. - SMN directly interacts with TRAF6 and the IKK complex in BV2 microglial cells [102].

Iba, ionized calcium-binding adaptor molecule 1; iNOS, inducible nitric oxide synthase; LPS, lipopolysaccharide; CD86, cluster of differentiation 86; SMA, spinal muscular atrophy; SMN, survival motor neuron; WT, wild-type; Mac-2, galectin-3; CD206, cluster of differentiation 206; P, postnatal day; CD68, cluster of differentiation 68; iPSC, induced pluripotent stem cell; GATA6, GATA binding factor 6; NFκB, nuclear factor kappa B; JNK, c-Jun N-terminal Kinase; IL-1β, interleukin-1 beta; IKK, inhibitor of nuclear factor-κB kinase; TNF-α, tumor necrosis factor alfa; NO, nitric oxide; TRAF6, tumor necrosis factor receptor associated factor 6; BV2, BV2 microglial cell line.

**Table 3:** Selected in vivo and in vitro studies on the role of oligodendrocytes in SMA.

In Vivo	
Model/Genotype	Notable Changes in Oligodendrocytes Morphology and Function
SMNΔ7mice [148]	- Oligodendrocyte lineages, including both OPCs and OLs, exhibit significant impairments in spinal cord of SMA mice [148]. - MBP (marker of mature OLs) and NG2 (OPC marker) expression levels are reduced in the spinal cords of SMNΔ7 mice compared to WT mice [148]. - Decrease in the NG2 expression is attributed to enhanced Notch signaling in spinal cord of SMA mice [148]. - Reduced NG2 expression impaired OPC proliferation in spinal cord SMA mice leading to the impaired OL differentiation [148]. - OL differentiation impairment occurred before lower MN degeneration [148].
Smn^−/−^; *SMN2* mice [27]	- The number of mature OLs in the corpus callosum of Smn^−/−^; *SMN2* mice was equivalent to WT controls, indicating that in vivo maturation is unaffected by SMN depletion [27]. - Electron microscopy revealed similar myelin structure in the spinal cords of Smn^−/−^; *SMN2* and WT mice, demonstrating intact myelination in the CNS [27].
Smn^−/−^; *SMN2*;SMNΔ7 mice [138]	Genetic changes in spinal cord oligodendrocyte development between P1 and P7, and P7 and P13 day old mice [138].
**In Vitro**	
**Model/Genotype**	**Notable Changes in Oligodendrocytes Morphology and Function**
SMA-iPSC culture containing motor neurons, astrocytes, and oligodendrocytes [148]	- Levels of MBP and Prox1 were significantly reduced in SMA-iPSC cultures [148]. - Reduced NG2 expression activates Notch signaling and suppresses Prox1 expression, resulting in impaired oligodendrocyte differentiation [148]. - Pharmacological inhibition of Notch signaling increased Prox1 expression in SMA-iPSC MNs and promoted MBP-positive OL differentiation [148].
Primary oligodendrocytes from Smn^−/−^; *SMN2* mice [27]	- Expression of OPC maturation (NG2) and differentiation (MBP) markers were similar in Smn^−/−^ *SMN2* and WT cultures, indicating that SMN levels do not affect OL differentiation [27]. - OPCs exhibited normal migratory behavior, successfully migrating to the same extent as WT counterparts [27]. - OLs morphological stages were comparable between genotypes at both DIV3 and DIV6, with no significant differences in complexity [27]. - OLs from Smn^−/−^ *SMN2* mice were equally capable of myelinating axons in co-culture systems, demonstrating no functional deficits in myelination [27].

iPSC, induced pluripotent stem cells; MBP, myelin basic protein; NG2, nerve/glial-antigen 2; OLs, mature oligodendrocytes; OPCs, oligodendrocyte precursor cells; SMA, spinal muscular atrophy; SMN, survival motor neuron; WT, wild-type; CNS, central nervous system; Prox1, prospero-related homeobox 1; P, postnatal day; DIV, days in vitro.

**Table 4:** Selected in vivo and in vitro studies on the role of Schwann cells in SMA.

In Vivo	
Model/Genotype	Notable Changes in Schwann Cells Morphology and Function
SMAΔ7, Hb9-GFP SMAΔ7, Smn^Res^/*SMN2*^+/+^/SMNΔ7^+/+^ expressing ChAT^Cre^, Dhh^Cre^ or MyoD^/Cre^ mice [173]	- Compromised motor axon radial expansion and deficient Schwann cell myelination in SMA mice started during embryonic development and led to compromising motor axon function neonatally [173]. - Postnatally, motor axons with impaired myelination quickly degenerated [173]. - A reduced number of Schwann cells reduces axon–Schwann cell sorting and radial axonal growth [173]. - Selective restoration of SMN protein in lower MNs significantly enhances axonal growth and stability, whereas restoring SMN in Schwann cells or muscle alone provides no measurable benefit [173].
Pharmacological SMA mice—SMN∆7 mice receiving splicing modifier SMN-C1 to generate mice with milder SMA phenotype [168]	The progressive loss of terminal Schwann cells, despite their retained ability to proliferate and migrate, contributes to disruptions in NMJ remodeling [168].
Smn^+/−^; *SMN2*^tg/0^, Smn^+/−^; *SMN2*^tg/0^; *SMN1*^SC^, Smn^−/−^; *SMN2*^tg/0^, Smn^−/−^; *SMN2*^tg/0^; *SMN1*^SC^ [174]	Transgenic SMA mice overexpressing SMN only in myelinating Schwann cells Smn^−/−^; *SMN2*^tg/0^; *SMN1*^SC^ corrected myelination defects, greatly enhanced neuromuscular function, and alleviated NMJ pathology [174].
Smn^+/−^; *SMN2*^+/+^ mice [170]	- Vacuole-like inclusions observed in the cytoplasm of terminal Schwann cells were associated with a loss of cellular identity due to the absence of the S100 cell marker [170]. - Alterations in the cytoplasm of terminal Schwann cells occur before other morphological changes, such as mitochondrial swelling in axon terminals and muscle fibers [170].
‘Severe’ SMA mouse model (Smn^−/−^; *SMN2*^tg/tg^) and ‘Taiwanese’ SMA mice (Smn^−/−^; *SMN2*^tg/0^) [175]	- Myelination deficits in intercostal nerves were evident during both early and late symptomatic phases of the disease in two distinct mouse models of SMA [175]. - Maturation of axo-glial interactions at paranodal regions was disrupted, while myelination of motor axons in the corticospinal tract of the spinal cord remained unaffected [175].
FVB.Cg-Tg(*SMN2*)89Ahmb *Smn1*tm1, smn^+/+^; hS*MN2*^+/+^ and B6d2F2xFVB/N mice [171,172]	Vacuole-like inclusions are present in terminal Schwann cells at NMJs with swollen mitochondria, particularly in the diaphragm and intercostal muscles.
*Smn^2B/−^* mice [167]	- A significant reduction in the number of terminal Schwann cells associated with each NMJ was identified [167]. - Despite this reduction, the remaining Schwann cells demonstrated preserved functionality in terms of their ability to migrate and proliferate [167].
SMAΔ7 mice [169]	A marked decrease in the number of terminal Schwann cells [169].
Smn^+/−^; *SMN2* and Smn^−/^; *SMN2*; delta7 mice [176]	Gene expression of laminin alpha-2 is increased at P1, which corresponds to the pre-symptomatic stage, but significantly decreases at P5 during the late-symptomatic stage, which suggests a disruption in extracellular matrix [176].
Smn^+/−^; *SMN2*; SMNΔ7 [138]	During the pre-symptomatic stage, at P1, increased gene expression in laminin alpha 2 is observed [138].
Smn^+/+^, Smn^+/−^, Smn^+/−^ Cntf^−/−^ and Smn^+/+^ Cntf^−/−^ mice [143]	- CNTF, which is highly expressed in myelinating Schwann cells, plays a crucial role in initiating a sprouting response [143]. - This process is essential for enhancing the size of motor units in skeletal muscles, thereby supporting motor function and plasticity [143].
**In Vitro**	
**Model/Genotype**	**Notable Changes in Schwann Cells Morphology and Function**
Primary Schwann cells from ‘Taiwanese’ SMA mice (Smn^−/−^; *SMN2*^tg/0^) [175]	- Following differentiation, cells fail to express key myelin proteins, likely due to disruptions in protein translation and/or stability rather than transcriptional defects [175]. - Co-culturing healthy neurons with affected Schwann cells revealed compromised myelination, pointing to inherent defects in the Schwann cells, as well as a decline in neurite stability [175]. - SMA Schwann cells were unable to express typical levels of essential extracellular matrix proteins, such as laminin α2 [175]. - The expression of myelin proteins in SMA Schwann cells was restored upon transfection with an SMN construct [175].
Primary Schwann cells from‘Taiwanese’ SMA mice (Smn^−/−^; *SMN2*^tg/0^) [145]	- SMN deficiency results in widespread alterations to the Schwann cell proteome, affecting pathways related to cell growth and proliferation, survival and cell death, and molecular transport [145]. - Significant disruptions in proteins involved in ubiquitination pathways, including a reduction in the levels of Uba1 [145]. - Pharmacological suppression of Uba1 replicated the differentiation and myelination defects seen in SMA Schwann cells with reduced SMN levels [145].

CNTF, ciliary neurotrophic factor; NMJ, neuromuscular junction; P, postnatal day; SMA, spinal muscular atrophy; SMN, survival motor neuron; Uba1, ubiquitin-like modifier activating enzyme 1; WT, wild-type.

## Abbreviations

The following abbreviations are used in this manuscript:

ALS	Amyotrophic lateral sclerosis
AMPA	α-amino-3-hydroxy-5-methyl-4-isoxazole propionic acid
ATP	Adenosine triphosphate
BCID	Bicaudal d
BDNF	Brain-derived neurotrophic factor
BV2	BV2 microglial cell line
CD206	Cluster of differentiation 206
CD68	Cluster of differ-entiation 68
CD86	Cluster of differentiation 86
CMT	Charcot-Marie-Tooth
CNS	Central nervous system
CNTF	Ciliary neurotrophic factor
DIV	Days in vitro
EAAT1	Excitatory amino acid transporter
ECM	Extracellular matrix
Erk1/2	Extracellular Signal-Regulated Kinases 1 and 2
GATA6	GATA-binding protein 6
GDNF	Glial-derived neurotrophic factor
GFAP	Glial fibrillary acidic protein
Iba1	Ionized calcium-binding adaptor molecule 1
IKK	Inhibitor of nuclear factor-κB kinase
IL-	Interleukin-
iNOS	Inducible nitric oxide synthase
iPSC	Induced pluripotent stem cell
JNK	c-Jun N-terminal Kinase
Kir4	Inwardly rectifying potassium channel 4.1
LAMA2	Laminin alpha 2
LPS	Lipopolysaccharide
MAPK	Mitogen-Activated Protein Kinase
MBP	Myelin basic protein
MN	Motor neuron
NfκB	Nuclear Factor kappa-light-chain-enhancer of activated B cells
NG2	Nerve/glial antigen 2
NLR	NOD-like receptor
NMJ	Neuromuscular junction
NO	Nitric oxide
OLs	Mature oligodendrocytes
OPC	Oligodendrocyte progenitor cells
PNS	Peripheral nervous system
Prox1	Prospero-related homeobox 1
PrP	Prion Protein Promoter
RGC	Radial glial cells
ROS	Reactive oxygen species
SMA	Spinal muscular atrophy
SMN	Survival motor neuron
TNF-α	Tumor necrosis factor alfa
TRAF6	Tumor necrosis factor receptor associated factor 6
TrkB	Tropomyosin receptor kinase B
WT	Wild-type
